# Comparative Gene Expression Analysis of Mouse and Human Cardiac Maturation

**DOI:** 10.1016/j.gpb.2016.04.004

**Published:** 2016-07-16

**Authors:** Hideki Uosaki, Y-h Taguchi

**Affiliations:** 1 Division of Cardiology, The Johns Hopkins University School of Medicine, Baltimore, MD 21205, USA; 2 Department of Physics, Chuo University, Tokyo 112-8551, Japan

**Keywords:** Cardiac maturation, Comparative gene expression analysis, Microarray meta analysis, Principal component analysis, Feature selection

## Abstract

Understanding how human cardiomyocytes mature is crucial to realizing stem cell-based heart regeneration, modeling adult heart diseases, and facilitating drug discovery. However, it is not feasible to analyze human samples for maturation due to inaccessibility to samples while cardiomyocytes mature during fetal development and childhood, as well as difficulty in avoiding variations among individuals. Using model animals such as mice can be a useful strategy; nonetheless, it is not well-understood whether and to what degree gene expression profiles during maturation are shared between humans and mice. Therefore, we performed a **comparative gene expression analysis** of mice and human samples. First, we examined two distinct mice microarray platforms for shared gene expression profiles, aiming to increase reliability of the analysis. We identified a set of genes displaying progressive changes during maturation based on **principal component analysis**. Second, we demonstrated that the genes identified had a differential expression pattern between adult and earlier stages (*e.g.*, fetus) common in mice and humans. Our findings provide a foundation for further genetic studies of cardiomyocyte maturation.

## Introduction

Pluripotent stem cells (PSCs) hold tremendous potential for regenerative medicine, disease modeling, and drug discovery in a broad spectrum of tissue and cell types, such as cardiomyocytes [1], [2], [3], [4]. Recent advances in the field have rendered efficient and robust differentiation of cardiomyocytes from most of PSC lines [5], [6], [7]. Although the maturation of differentiated cardiomyocytes into the adult-like stage is essential to study adult-onset diseases *in vitro*, fully matured cardiomyocytes have never been obtained [8]. Moreover, there are no clear-cut and definitive markers available to evaluate cardiomyocyte maturation [8]. Therefore, a detailed understanding of the cardiac maturation process *in vivo* is a prerequisite for further development of methods to maturate PSC-derived cardiomyocytes *in vitro*.

Uosaki et al. examined the detailed process of mice cardiac maturation using meta-microarray analysis [9]. This and other studies demonstrated that the maturation of cardiomyocytes is a continuous process occurring during embryonic and postnatal development [9], [10], [11], [12]. Because of limited human samples obtained during the early life (potentially collected from aborted fetus, babies that died from accidents or other medical reasons, and/or biopsies from transplanted hearts) and technical difficulty in repetitive sample collection from the same individual, it is difficult to dissect the progression in humans from individual variations, *e.g.*, by measuring gene expression. Therefore, studies of cardiac maturation rely heavily on model animals, *e.g.*, mice. Here, the key question remain to be addressed is whether and to what extent cardiac maturation progresses are similar in mice and humans.

Comparative gene expression analysis [13] is a useful strategy to evaluate consistency between species. It enables studying multiple human diseases in mice, which are hard to investigate directly in humans [14]. It can even help us to understand gene regulatory mechanisms in mammals using gene expression data from non-mammalian animals [15]. Moreover, it also helps in identifying highly-correlative expression profiles between putative orthologs across species [16].

In this study, we demonstrated the correlation of gene expression involved in cardiac maturation between mice and humans. We performed a meta-microarray analysis of data generated from mice samples ranging from the embryonic to the adult stages using two microarray platforms (Affymetrix Mouse Genome 430 2.0 Array, referred to as “mouse 430 2.0” hereafter and Mouse Gene 1.0 ST Array, referred as “mogene 1.0” hereafter) to collect a reliable set of genes correlating with the progression of cardiac maturation in mice. Subsequently, we evaluated whether highly-correlative expression profiles that were identified in the mice gene set exist in human samples.

## Results

### Performance comparison between frozen robust microarray analysis and microarray suite 5 method

In our previous paper [9], we employed the frozen robust microarray analysis (fRMA) [17] to analyze the gene expression profiles of more than 200 microarray datasets ranging from early embryonic to adult hearts. fRMA serves as a reliable platform to perform meta-microarray analysis [17]. Nonetheless, fRMA can only be applied to popular microarray platforms, such as mouse 430 2.0 and mogene 1.0, due to its requirement of preprocessed dataset. In addition, there is uncertainty on whether fRMA correctly performs batch effect extraction, although this is one of the primary reasons why fRMA is introduced. On the other hand, microarray suite 5 method (MAS5) is a method used for single-microarray preprocessing [18]. We hypothesized that MAS5 can replace fRMA for meta-microarray analysis.

**Figure 1:**
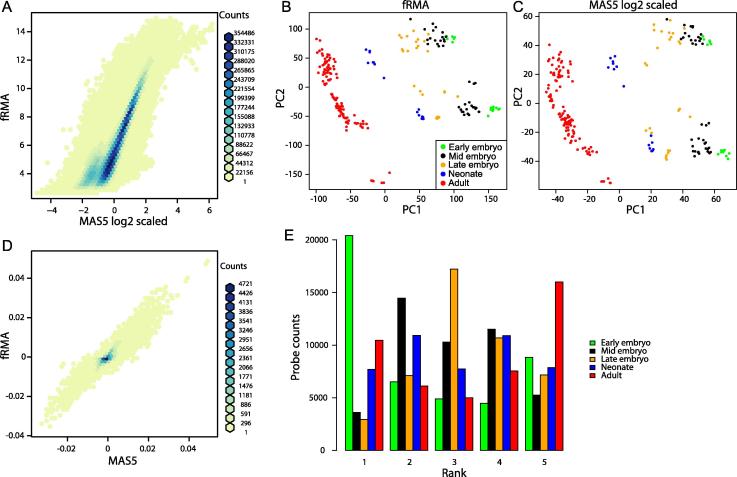
**Comparison of MAS5 and fRMA for mouse 430 2.0 array data preprocessing** **A.** Hex Bin scatter plot of individual probe sets, obtained from 646 microarray and 45,101 probesets. MAS5 preprocessed, log2-converted, and scaled data are presented on *X*-axis, whereas fRMA-preprocessed data are presented on *Y*-axis. The number of probesets in each hex bin is color-coded as shown in the figure. Scatter plots are presented for PC scores of PCA for fRMA-preprocessed data (**B**) and MAS5-preprocessed, log2-converted and scaled data (**C**). Samples at each developmental stage are represented in green (early embryo), black (mid embryo), orange (late embryo), blue (neonate) and red (adult), respectively. **D.** Hex Bin scatter plot of variable loadings for PC1 in PCA with MAS5- and fRMA-preprocessed data. The number of probesets in each hex bin is color-coded as shown in the figure. **E.** Histograms of ranked probe counts for each developmental stage. fRMA, frozen robust microarray analysis. PCA, principal component analysis.

To evaluate the performance of MAS5 for data preprocessing, we collected 646 microarray datasets (Table S1) and preprocessed them with MAS5 as well as fRMA. To allow comparison, MAS5-processed data was log2 transformed and scaled (mean = 0; standard deviation = 1). Signal intensities of all 45,101 probesets on mouse 430 2.0 platform were well correlated between MAS5 and fRMA (*R* = 0.90; Pearson correlation) (Figure1**A**). Although probes with medium signal intensities (6–12 in fRMA) showed better correlation, more variability was observed for probes with lower or higher signal intensities. To evaluate whether this variability would compromise the overall analysis, we conducted principal component analysis (PCA) for signal intensities of preprocessed data by fRMA (Figure1**B**) and MAS5 (Figure1**C**). The scatter plots of the first and second principal component (PC1 and PC2) values were almost identical. In addition, variable loadings for PC1 were well correlated between data preprocessed by fRMA and MAS5 (*R* = 0.89; Pearson correlation) (Figure1**D**). These results suggest that MAS5 can replace fRMA for meta-microarray analysis. Therefore, data preprocessed by MAS5 were used for downstream analyses. As pointed out previously [9], PC1 represents the maturation process and PC2 seems to separate batch effects in either preprocessing method.

As PCA indicated a gradual maturation process in the heart [9], we next assessed how gene expression changes during the maturation process. To detect gross changes, we averaged the signal intensities of each probe at each developmental stage for ranking. Figure1**E** depicts the distribution of the intensity ranks. As expected, the majority of probesets at the early embryonic and adult stages ranked either first or fifth, whereas more than one third of the probes at the late embryonic stage ranked third, suggesting that the expression of each gene changes gradually and unidirectionally. This finding is important when considering the limited datasets of human heart samples, which are mostly early-gestation fetal and adult samples, for comparative genomics.

### Probe–gene conversion

**Figure 2:**
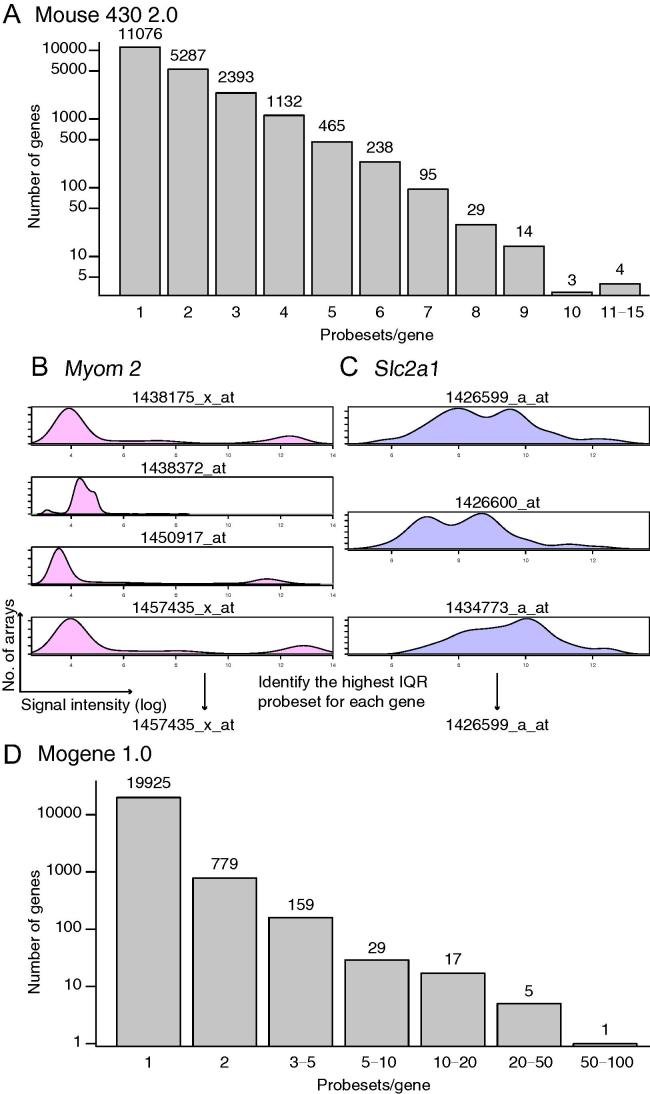
**Probeset-to-gene symbol conversion** **A.** Histogram showing the numbers of probesets annotated to single genes on the mouse 430 2.0 array. *X*-axis is numbers of probesets per gene and *Y*-axis is shown in logarithmic scale. Distribution of signal intensities and numbers of arrays for each probeset corresponding to *Myom2* (**B**) and *Slc2a1* (**C**), respectively. *X*-axis indicates the log2-transformed signal intensity of gene expression, whereas the numbers of arrays for the genes are shown on *Y*-axis. **D.** Histogram showing the numbers of probesets annotated to single genes on the mogene 1.0 array. *X*-axis is numbers of probesets per gene and *Y*-axis is on the logarithmic scale. IQR, interquartile range; Myom2, myomesin 2; Slc2a1, solute carrier family 2 (facilitated glucose transporter), member 1.

To perform comparative gene expression analysis, it is necessary to convert probesets to genes. In mouse 430 2.0, there were more than 45,000 probesets for 20,736 genes. We used mouse 4302.db to annotate probesets to genes. As a result, 11,076 genes were annotated to single probesets, whereas the remaining genes were annotated to at least two probesets (Figure2**A**). Seita et al. reported that identifying probes with the most dynamic ranges can be a good way to select probes [19]. However, such a method might be vulnerable to noise. Therefore, we decided to choose probes based on the interquartile ranges (IQRs) rather than the full dynamic ranges. For instance, *myomesin 2* (*Myom2*), encoding an M-protein that is expressed in mature cardiomyocytes [20], was annotated to 4 different probesets (Figure2**B**). One probeset (1438372_at) showed a very small dynamic range, whereas the other three probesets displayed similar but distinct patterns, with the widest IQR observed for the 1457435_x_at probeset. Different from *Myom2*, *Slc2a1*that encodes glucose transporter 1 (Glut1) was annotated to 3 probesets (Figure2**C**), which share similar IQRs. In contrast to mouse 430 2.0, more than 95% (19,925 out of 20,915 in total) of genes were annotated to a single probeset in mogene 1.0 when using mogene10sttranscriptcluster.db to annotate probesets to genes (Figure2**D**). Therefore, for the mogene 1.0 data, we simply averaged the signal intensities from multiple probesets to obtain the expression level of a particular gene.

### Identification of mice genes associated with cardiac maturation using PCA

**Figure 3:**
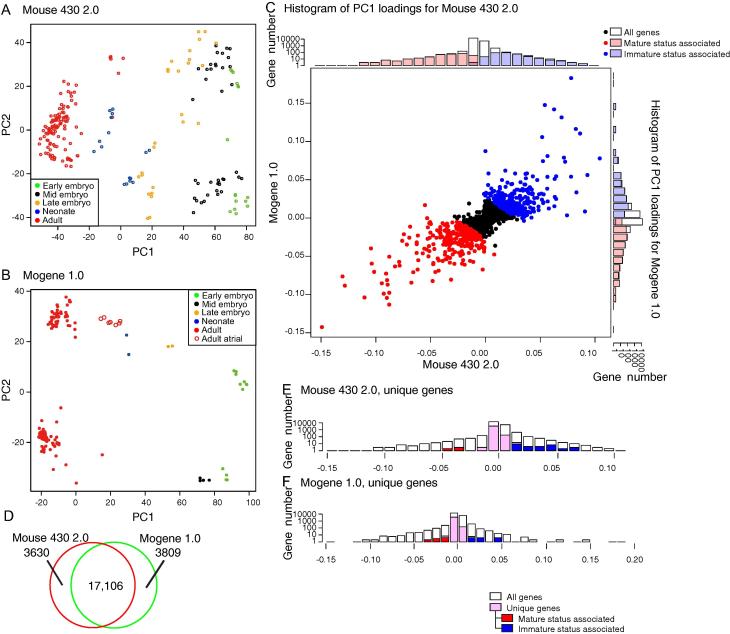
**Selection of genes associated with cardiac maturation** PCA scatter plots for all genes in mouse 430 2.0 (**A**) and mogene 1.0 arrays (**B**) are shown. Samples at each developmental stage are represented in green (early embryo), black (mid embryo), orange (late embryo), blue (neonate) and red (adult), respectively. Open circles in panel B represent adult atrial samples. **C.** Scatter plots and histograms for PC1 loadings of genes for mouse 430 2.0 (*X*-axis) and mogene 1.0 (*Y*-axis) data. PC1 loadings of each gene from both arrays were added and genes outside of the mean ± 2SD of summed PC1 loadings were selected as maturation-associated genes. Red and blue spots represent genes associated with mature status (mean − 2SD) and immature status (mean + 2SD), respectively, whereas as genes with expression changes between mean + 2SD and mean − 2SD are indicated in black. Histograms on *X*- and *Y*-axes depict the distribution of selected genes in the respective arrays. Gene numbers are shown on a logarithmic scale. **D.** Venn diagram of annotated genes on the mouse 430 2.0 and mogene 1.0 arrays. Histograms of PC1 loadings of unique genes on the mouse 430 2.0 or mogene 1.0 arrays are shown in panels **E** and **F**, respectively. Genes with mean ± 2SD of respective PC1 loadings are colored in red and blue.

Next, we used PCA to identify genes associated with cardiac maturation in mice. As shown in Figure1C with probe-level PCA, PCA clearly distinguished the samples from different stages (Figure3**A**). Neonatal samples were grouped into two clusters. Notably, one neonatal cluster close to the late embryonic stage and the other cluster close to the adult stage included samples from postnatal day (P)3 and P7, respectively, supporting the notion that PC1 is an explanatory variable for cardiac maturation. Similarly, we also performed PCA for the mogene 1.0 data (Figure3**B**). For some unknown reasons, data for some samples from a single institute were widely divergent from the other datasets. Therefore, these samples were excluded from entire analysis (data not shown, marked as “GSI” in Table S2). Although the number of samples for each stage was small and plots were sparse, the overall patterns for PCA plots were similar between the mouse 430 2.0 array data and mogene 1.0 data.

To identify genes associated with cardiac maturation, we first plotted PC1 loadings of each gene for mouse 430 2.0 and mogene 1.0 data (Figure3**C**). The loadings were well correlated (*R* = 0.78). Next, we added the individual loadings for each gene. As the summed loadings followed a normal distribution (data not shown), we selected genes with loadings higher than mean + 2 standard deviation (SD) and lower than mean - 2SD as genes that are significantly associated with cardiac maturation (colored in blue and red, respectively, in Figure3C). As more than 3600 genes were unique to either array (Figure3**D**), we also determined significant genes for each of the two arrays (Figure3**E** and **F**). In total, we identified 648 genes, including 293 and 355 genes associated with mature and immature status, respectively (full lists available in Table S3).

### Characterization of the maturation-associated genes

**Figure 4:**
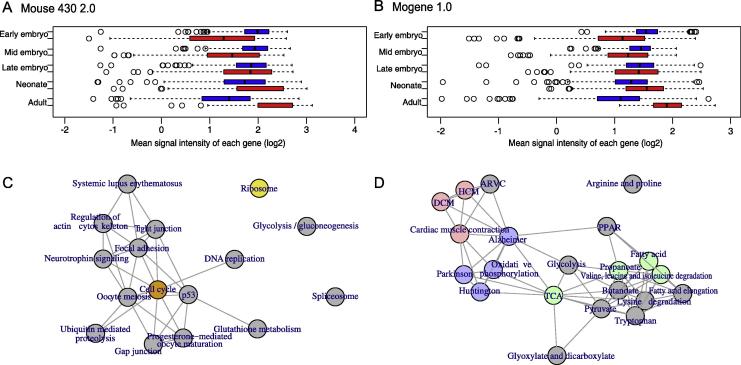
**Characterization of the genes associated with cardiac maturation** Box plots of mean signal intensities of each gene at different developmental stages for mouse 430 2.0 or mogene 1.0 arrays are shown in panels **A** and **B**, respectively. Red and blue boxes indicate the maturation-associated and immature stage-related genes, respectively. KEGG pathway networks for genes associated with immature status and mature status are shown in panels **C** and **D**, respectively. Colored nodes indicate FDR < 0.01, while gray nodes indicate FDR > 0.01 among the KEGG pathway enriched in the identified genes. Nodes are colored as follows: cardiac (red), oxidation (blue), mitochondria function and metabolism (green), cell cycle (orange), ribosome (yellow). DCM, dilated cardiomyopathy; HCM, hypertrophic cardiomyopathy; ARVC, arrhythmogenic right ventricular cardiomyopathy; TCA, tricarboxylic acid cycle; PPAR, peroxisome proliferator-activated receptor.

A linear model was employed to examine whether the genes identified above followed the trajectory of maturation (Figure1E). First, we averaged the signal intensities of genes across samples of certain stages, which changed gradually with progressing stages for both mouse 430 2.0 (Figure4**A**) and mogene 1.0 (Figure4**B**). We next conducted the linear regression analysis for each gene to obtain *P* values and calculated false discovery rates (FDRs) in order to adjust for multiple comparisons. Approximately 98% and 89% of the identified genes in the mouse 430 2.0 and mogene 1.0 arrays, respectively, had an FDR < 0.10, suggesting linear gene expression alterations for most of the genes identified.

To further characterize biological properties of the identified genes, we performed KEGG pathway analysis with DAVID [21], [22]. Pathways with an FDR < 0.01 were considered significant (nodes in color, Figure4**C** and **D**, Table S4 and S5). For the genes associated with immature status, ribosome- and cell cycle-related (*e.g.*, DNA replication and oocyte meiosis) pathways were significantly enriched (mmu03010: ribosome; mmu04110: cell cycle, Figure4C, Table S4). On the other hand, for the genes associated with mature status, oxidation and mitochondria-related pathways (mmu05012: Parkinson’s disease; mmu00190: oxidative phosphorylation; mmu05010: Alzheimer’s disease; mmu05016: Huntington’s disease; mmu00020: citrate cycle or TCA cycle) and cardiac pathways (mmu04260: cardiac muscle contraction; mmu05414: dilated cardiomyopathy, DCM, and mmu05410: hypertrophic cardiomyopathy (HCM)) were significantly enriched (Figure4D, Table S5). Taken together, these findings indicate that the genes identified are associated with cardiac maturation.

### Comparison with human datasets

**Figure 5:**
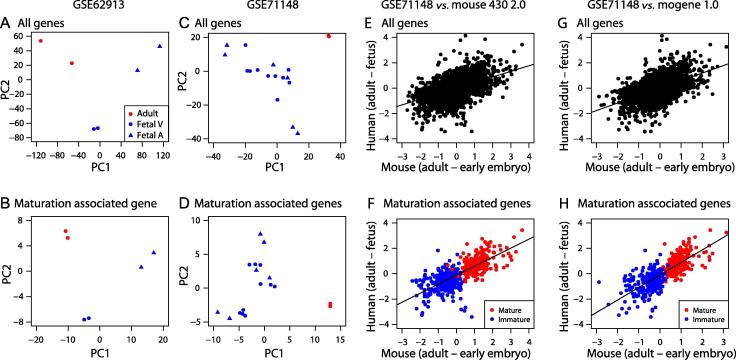
**Comparison of mice datasets with human datasets** Scatter plots for PC scores of different datasets are generated with all genes or the maturation-related genes. Scatter plots for GSE62913 using all genes or just the maturation-related genes are shown in panels **A** and **B**, respectively, while for GSE71148, **C** and **D** indicate the plots generated using all genes or the maturation-related genes, respectively. Adult heart samples are indicated with red circle, while fetal ventricle (Fetal V) and fetal atrium (Fetal A) samples are indicated with circle and triangle in blue, respectively. Scatter plots for expression changes between human GSE 71148 and mice datasets are shown in panels **E**–**H** using all genes or just maturation-associated genes. Comparison between GSE 71148 and mouse 430 2.0 was conducted using all genes (E) or maturation-related genes (F). Similarly, comparison between GSE 71148 and mogene 1.0 was conducted using all genes (G) or maturation-related genes (H). Differential signal intensities for gene expression between early embryonic/fetal stage and adult stage were used. Linear regression lines are also plotted. X axes indicate gene expression changes (differences of log2-transformed scaled signal intensities) from early embryonic to adult stages in the respective mice arrays, while Y axes indicate gene expression changes from fetus to adult stage in human GSE71148.

Finally, we assessed the expression patterns of the genes identified in mice in human datasets. We found two distinct datasets of human hearts including fetal and adult hearts (GSE62913 and GSE71148) [22], [23]. GSE62913 contains RNA-seq data obtained from fetal ventricles and atria, as well as adult hearts. We performed PCA with all genes as well as with the maturation-associated genes, respectively. Among the 648 maturation-associated mice genes identified above, we found human counterparts of 520 genes in the GSE62913 dataset (234 and 286 for mature and immature status, respectively). PCA with all genes as well as with maturation-associated genes similarly revealed distinctive patterns between fetal samples and adult hearts (Figure5**A** and **B**). The other dataset GSE71148 is an Illumina HumanHT-12 V4.0 expression beadchip dataset for fetal and adult heart samples. We identified 586 maturation-associated genes conserved between humans and mice (262 and 324 for mature and immature status, respectively). Consistent with the PCA on GSE62913, PCA on GSE71148 with all genes or the maturation-associated genes both generated patterns distinctive between fetal and adult samples (Figure5**C** and **D**).

To assess whether gene expression patterns in mice and humans are correlated and whether the usage of maturation-associated genes improves the correlation over the usage of all genes, we compared expression changes in mice and humans using all genes or the maturation-associated genes only (Figure5**E–H**). As the human fetal heart samples were from fetus in the first and second trimesters (7–20 weeks), we used early embryonic mice hearts for comparison. We found that expression changes between adult and early embryo/fetus using all genes showed good correlation between mice and humans for mouse 430 2.0 dataset (*R* = 0.49, Figure5E) and mogene 1.0 dataset (*R* = 0.51, Figure5G). Nonetheless, the gene expression changes of maturation-related genes alone showed better correlation for both datasets (*R* = 0.73 for mouse 430 2.0, Figure5F and *R* = 0.78 for mogene 1.0, Figure5H). Overall, 286 out of 324 immature status-associated genes and 237 out of 262 mature status-associated genes showed higher expression in fetal and adult hearts, respectively. Interestingly, most of the genes that showed inconsistency with the findings in mice did not show significant differences between fetal and adult heart samples in humans (only 8 genes showing more than 1.5-fold changes, Table S6). It is of note that *MYH7* was among the immature-associated genes identified in the mice, and was highly expressed in human adult hearts as is widely known.

Taken together, gene expression pattern of cardiac maturation between early embryonic/fetal and adult stages is mostly consistent across species, and the maturation-related genes identified in mice can be mostly recapitulated in humans.

## Discussion

In this study, we identified cardiac maturation-associated genes in mice based on PCA of data from two distinct mice microarrays. We demonstrated that the expression of the genes identified change progressively during maturation and that the expression patterns are well conserved between mice and humans. Although mice and human adult cardiomyocytes are different in terms of cell size, length of action potential, and beating rate, *etc.*, they share some common features *e.g.*, morphology, abundant mitochondria, and sarcomere structure [8]. Our findings indicate that mice and humans follow a similar maturation process. *MYH6* and *MYH7*, the genes encoding alpha and beta myosin heavy chains, are differentially expressed in mice and humans. *Myh6* encodes a predominant form of myosin heavy chain in adult mice heart and *Myh7* is expressed in embryonic mice heart, whereas opposite expression pattern of these two genes is found in humans [24], [25]. In accordance herewith, our comparative gene expression analysis successfully identified that *MYH7* is a gene associated with immature stage in mice, but highly upregulated in human adult hearts.

Cells derived from either mice model or mice/human PSCs are often used for maturation studies. However, PSC-derived cardiomyocytes barely mature [9]. More importantly, there are no established readouts to define maturation status of cardiomyocytes. Structural and functional readouts, which include cell size, morphology, t-tubule formation, calcium handling, action potential, and mitochondrial function, are often used [26], [27], [28]. It is known how morphology and structure change during maturation in mice or rat but it is unknown for human. Physiological features were only studied for adult cardiomyocytes but not for embryonic and neonatal cardiomyocytes. Therefore, these readouts cannot be used to measure maturation status quantitatively at this point. The gene list we provided (Table S3) could serve as a resource for developing defined, objective, and reliable readouts, as expression of these genes change monotonically during maturation in both mice and humans.

As we used PCA-based gene selection and made a comparison only between the adult and early embryonic/fetal stages, some of the highly differentially-expressed genes shown in Figure5E and G were not selected based on PCA. Thus, we took an alternative approach for gene selection to evaluate whether the genes that are highly differentially expressed between adult and early embryo/fetus are sufficient to recapitulate the heart maturation pattern. Briefly, we summed the human and mice differential signal intensities of each gene. As the summed differential signal intensities followed a normal distribution, we selected genes for which expression levels fell out of the range of mean ± 2SD (Figure S1A and S1B). Although only one third of the alternatively selected genes overlapped with the genes selected using the PCA-based method (Figure S1C and S1D), the PCA patterns generated with the alternatively selected genes were similar to those generated with all genes (Figure S1E–H). As we demonstrated in Figure1E as well as Figure4A and B, the maturation process in the heart is unidirectional, and most genes related to maturation changed progressively. Therefore, the genes highly differentially expressed between the adult and early embryoic/fetal stages successfully represented the maturation process, which would be more appropriate for finding specifically-expressed genes. PCA granted unidirectional change and would be more appropriate for studying the process of maturation.

Finally, in this study, we also tackled a bioinformatics issue—the limitations of fRMA. Although fRMA was designed to avoid batch effects by using frozen data sets generated from a large quantity of datasets, fRMA did not outperform MAS5, which is a single array-based normalization method. Our results demonstrate that the performance of fRMA is correlated well with that of MAS5, suggesting that MAS5 can be used in place for fRMA.

## Conclusions

In this study, we performed a comparative gene expression analysis of mice and human cardiac maturation. As a result, we identified more than 500 genes that share distinct expression patterns during cardiac maturation between mice and humans. These genes could be further explored for their potential as genetic markers to investigate cardiomyocyte maturation in future.

## Methods

### mRNA expression

All mRNA expression profiles analyzed in this study were downloaded from the Gene Expression Omnibus (GEO, http://www.ncbi.nlm.nih.gov/geo/). Mouse 430 2.0 gene expression profile was selected from profiles analyzed in our previous study [9]. Detailed information about mouse 430 2.0 and mogene 1.0 arrays is listed in Tables S1 and S2, respectively. Profiles analyzed in Figure 1, Figure 3, Figure 4 were generated from five developmental stages with sample numbers (N) provided for mouse 430 2.0 and mogene 1.0, respectively. These include early embryonic (embryonic day (E)8–11, *N* = 16 and 12), mid embryonic (E12–15, *N* = 39 and 4), late embryonic (E16–18, *N* = 26 and 2), neonate (postnatal day (P)1–10, *N* = 16 and 2), and adult (>4-week old, *N* = 115 and 134) stages. Only wild-type and non-treated samples were included in the current study. Human gene expression profiles were taken from GSE62913 and GSE71148. GSE62193 contains RNA-seq data for human PSC-derived cells, as well as fetal and adult hearts, whereas GSE71148 comes from an Illumina array transcriptome study for 20 samples from fetal and adult hearts, including Ref-pool (GSM1828516).

### Preprocessing

Multiple preprocessing methods were employed in this study. “MAS5-scale” indicates scaling was performed after MAS5 preprocessing, while “MAS5-log2-scale” indicates that a log2 transformation was performed before scaling but after MAS5 preprocessing.

#### fRMA

fRMA was conducted using the Bioconductor/R fRMA package. Annotation packages mouse4302frmavecs and mogene.1.0.st.v1frmavecs were used for mouse 430 2.0 and mogene 1.0 arrays, respectively.

#### MAS5

MAS5 normalization was conducted for mouse 430 2.0 and mogene 1.0 data by using the MAS5 function in the Bioconductor/R affy and xps packages, respectively.

#### Scaling and log2 transformation

Scaling, which extract means and normalize standard deviation to one, was performed with the scale function in R. Additionally, log2 transformation was also performed using R.

#### Probeset–gene conversion

To convert probesets to genes, we identified probesets with the highest IQRs of signal intensity for mouse 430 2.0. To determine the IQR, we analyzed 429 arrays for brain, 212 arrays for heart, 142 arrays for kidney, and 137 arrays for liver. All arrays were preprocessed with fRMA and the IQR was determined for each probeset. The probe*–*gene match list was used to convert MAS5-preprocessed data. The conversion table is available as Table S7. As only less than 5% of genes were annotated to multiple probesets in mogene 1.0 (Figure2D), we simply averaged the signal intensities of multiple probesets for a particular gene.

#### Human datasets

Read count data of GSE62193 were scaled to normalize the individual samples (mean = 1 and standard deviation = 0), while normalized and log2-transformed data for GSE71148 was directly obtained from GEO and used for subsequent analysis.

#### PCA

PCA was conducted using the prcomp function in R to demonstrate overall differences of samples.

### Identification of maturation-associated genes

Maturation-associated genes were identified using two different approaches. For genes common to the mouse 430 2.0 and mogene 1.0 arrays, PC1 loadings of each array were summed. Genes with summed PC1 loadings more than a mean + 2SD or less than a mean − 2SD were selected as maturation-associated genes. On the other hand, for genes unique to either of arrays, genes with PC1 loadings more than a mean + 2SD or less than a mean − 2SD of the corresponding array were selected.

### Developmental stage wide coarse-grained gene expression analysis

In this analysis, we employed MAS5 preprocessed profiles generated from the mouse 430 2.0 array. Average of expression of the *i-*th gene at each developmental stage, *x_is_*, was defined as $x_{\mathrm{is}}\equiv \frac{1}{N_{s}}\sum_{j\in s}x_{\mathrm{ij}}$ where *s* is one of five aforementioned developmental stages and *N_s_* is the number of samples that belong to the stage, *x_ij_* is expression of the *i*-th gene in *j*-th samples. Averaged values were subsequently ranked across stages.

### Linear regression analysis of developmental-stage coarse-grained gene expression

Regression analysis was done using the following equation: *x_is_* = *a_i_*s + *b*_i_, where *a_i_* and *b_i_* are the regression coefficients, and *s* takes values 1–5 corresponding to the developmental stages in the order of early, mid, late, neonatal, and adult, respectively. The linear regression analysis was carried out using lm function in R [29]. *P* values were adjusted to meet FDR criterion using the fdrtool function in the fdrtool [30] package. Regressions with *q* values (adjusted *P* values) <0.1 were regarded to be significant.

### KEGG pathway enrichment analysis

Enrichment analysis for KEGG pathways was performed by uploading gene symbols to DAVID. Numbers of genes overlapping between KEGG pathways were used as weights to generate KEGG pathway networks shown in Figure4C and D with the igraph [31] package in R [29].

### Mapping of mice genes to human genes

Identical official gene symbols found in mice and human data were considered as a pair and used for comparison in Figure 5.

## Authors’ contributions

HU and YHT planed the research project; HU performed all analyses. Both HU and YHT were involved in manuscript writing, read and approved the final manuscript.

## Competing interests

The authors have declared that there are no competing interests.
