# Supplementary figures and images for: Comparative Gene Expression Analysis of Mouse and Human Cardiac Maturation
Source: Genomics Proteomics Bioinformatics. 2016 Jul 16;14(4):207–15. doi: 10.1016/j.gpb.2016.04.004 (PMC4996857; doi:10.1016/j.gpb.2016.04.004)

**A** Mouse 430 2.0 vs. GSE71148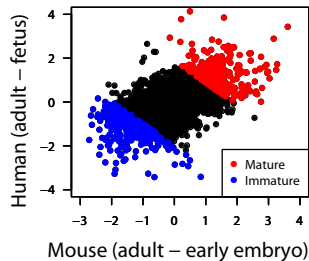**C** Mouse 430 2.0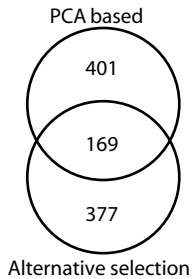**E** Mouse 430 2.0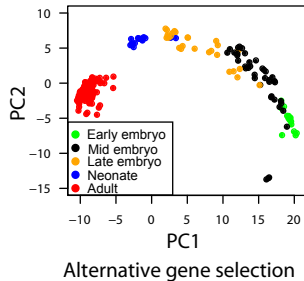**G** Mogene 1.0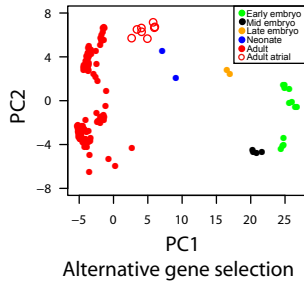**B** Mogene 1.0 vs. GSE71148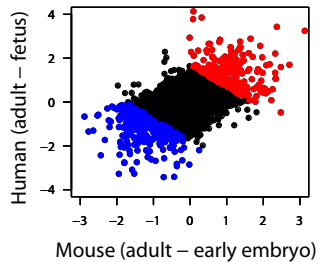**D** Mogene 1.0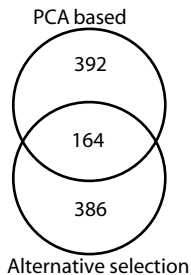**F** Mouse 430 2.0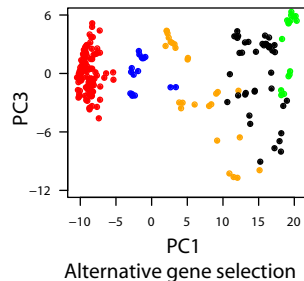**H** GSE71148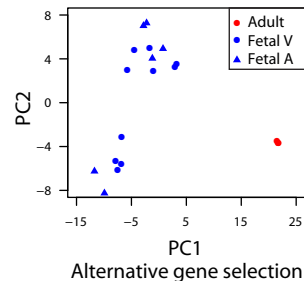

Supplement: Supplementary Figure 1 [file mmc1.pdf]
